# Cell Specific Post-Translational Processing of Pikachurin, A Protein Involved in Retinal Synaptogenesis

**DOI:** 10.1371/journal.pone.0050552

**Published:** 2012-12-04

**Authors:** Jianzhong Han, Ellen Townes-Anderson

**Affiliations:** Department of Neurology and Neuroscience, New Jersey Medical School, University of Medicine and Dentistry of New Jersey, Newark, New Jersey, United States of America; Dalhousie University, Canada

## Abstract

Pikachurin is a recently identified, highly conserved, extracellular matrix-like protein. Murine pikachurin has 1,017 amino acids (∼110 kDa), can bind to α-dystroglycan, and has been found to localize mainly in the synaptic cleft of photoreceptor ribbon synapses. Its knockout selectively disrupts synaptogenesis between photoreceptor and bipolar cells. To further characterize this synaptic protein, we used an antibody raised against the N-terminal of murine pikachurin on Western blots of mammalian and amphibian retinas and found, unexpectedly, that a low weight ∼60-kDa band was the predominant signal for endogenous pikachurin. This band was predicted to be an N-terminal product of post-translational cleavage of pikachurin. A similar sized protein was also detected in human Y79 retinoblastoma cells, a cell line with characteristics of photoreceptor cells. In Y79 cells, endogenous pikachurin immunofluorescence was found on the cell surface of living cells. The expression of the N-fragment was not significantly affected by dystroglycan overexpression in spite of the biochemical evidence for pikachurin-α-dystroglycan binding. The presence of a corresponding endogenous C-fragment was not determined because of the lack of a suitable antibody. However, a protein of ∼65 kDa was detected in Y79 cells expressing recombinant pikachurin with a C-terminal tag. In contrast, in QBI-HEK 293A cells, whose endogenous pikachurin protein level is negligible, recombinant pikachurin did not appear to be cleaved. Instead pikachurin was found either intact or as dimers. Finally, whole and N- and C-fragments of recombinant pikachurin were present in the conditioned media of Y79 cells indicating the secretion of pikachurin. The site of cleavage, however, was not conclusively determined. Our data suggest the existence of post-translational cleavage of pikachurin protein as well as the extracellular localization of cleaved protein specifically by retinal cells. The functions of the pikachurin N- and C-fragments in the photoreceptor ribbon synapse are unknown.

## Introduction

Understanding the formation and function of the first synapse in the visual pathway- the tripartite ribbon contact between photoreceptors and the second order horizontal and bipolar neurons- is critical to understanding visual processing. Moreover, the integrity of this first synapse is essential since without it almost all vision is lost even if the rest of the visual pathway remains intact. Pikachurin was recently identified as a highly conserved extracellular matrix (ECM)-like protein with a molecular weight around 110 kDa and high mRNA abundance in retina [1], [2]. As seen by immunocytochemistry, pikachurin is present in the synaptic cleft of photoreceptor ribbon synapses in adult murine retina and most importantly its absence specifically disrupts the apposition of bipolar cell dendrites to photoreceptor terminals [1]. Finally, pikachurin has been reported to bind to α-dystroglycan (α-DG) and this interaction has been suggested to contribute to its function in the retina [1]. The characterization of pikachurin, however, is incomplete. Notably, there are to date no Western blot analyses. Thus, although it has been suggested that pikachurin links bipolar cell dendrites to photoreceptors [1], many questions remain.

In this study, we began by examining endogenous pikachurin from vertebrate retinas with Western blot analysis. The protein appears more complex than first suggested. We report that the majority of pikachurin in adult retina is post-translationally cleaved resulting in an N-terminal fragment of 60 kDa. In a human retinoblastoma cell line, Y79, the N-terminal fragments were produced from both endogenous and recombinant pikachurin, and were found on the extracellular surface; a C-terminal fragment was additionally demonstrated using recombinant protein. Moreover, when recombinant pikachurin was expressed in Y79 cells, the products of pikachurin cleavage, the N- and C-fragments, along with the whole protein were present in the conditioned medium and apparently highly glycosylated. Our findings suggest that there is post-translational modification of the pikachurin protein in the retina, which may have unique, but as yet unknown, importance to the function of the photoreceptor-bipolar synapse. These results have been previously reported in part at meetings of the Association for Researchers in Vision and Ophthalmology (ARVO).

## Materials and Methods

### Ethics Statement

Salamanders and mice were maintained in a central animal facility under pathogen-free conditions. All animal protocols were approved by the UMDNJ Institutional Animal Care and Use Committee.

### Antibodies

The polyclonal antibody against pikachurin [1] was provided by Dr. T. Furukawa (Osaka Bioscience Institute, Japan) in early experiments and purchased from Wako Chemicals USA after it was commercialized. Other primary antibodies used were: c-Myc mAb (Sigma), His-tag pAb (Cell Signaling Technologies), His-tag mAb, α-DG mAb, GFP mAb, GFP pAb, GAPDH mAb (Santa Cruz Biotechnology, Inc.), α-DG mAb (Millipore) and β-DG mAb (Developmental Studies Hybridoma Bank, University of Iowa). Secondary antibodies were conjugated to either Alex Fluor (Invitrogen) or horseradish peroxidase (HRP; Jackson ImmunoResearch Laboratories).

### Retinas

Human retinal tissue was from donor eyes and was provided by Dr. M. Zarbin (New Jersey Medical School- UMDNJ) [3]. Adult porcine eyes were purchased from a local slaughterhouse (Animal Parts, Scotch Plains, NJ), delivered on ice, and retinas were harvested as previously described [4] within 2 hours of death. Salamander retinal tissue was obtained from adult aquatic-phase tiger salamanders and isolated as previously described [5], [6].

### Cells

Y79 human retinoblastoma cells (ATCC) were grown in suspension in RPMI 1640 supplemented with 10–20% FBS at 37°C with 5% CO2. Before experiments, Y79 cells were plated on poly-L-lysine (Sigma, >300 kDa) at appropriate density to form a monolayer and maintained in DMEM with 2% FBS (D2 Media). In some cases, Y79 cells were cultured in serum free media (N2 media) consisting of DMEM, 10 µg/ml transferrin (Sigma), 5 µl/ml insulin (Sigma), 5 ng/ml sodium selenite (Sigma), 7.3 ng/ml progesterone (Sigma), and 16 µg/ml putrescine (Sigma). To block protease activity in the culture media, a protease inhibitor cocktail (Sigma, P1860, 1∶400 dilution) was included in the media until cell lysates and conditioned media were prepared. QBI-HEK 293A cells (Qbiogene) were cultured in DMEM supplemented with 10% FBS at 37°C with 5% CO2.

### RT-PCR

The PureLink RNA mini kit (Invitrogen) was used to purify total RNA from cultured cells. Pikachurin mRNA expression was examined by RT-PCR (Qiagen OneStep RT-PCR kit). Primers used in the reactions were: forward primer (ACTCCATGGTTATCAAGGGCC), reverse primer (AGGCCAGCTGGTGTTACTTGGC). The PCR product was predicted to be 2472 kb, encompassing most of the encoding sequence of the longest human pikachurin variant (GenBank # NM_001205301).

### Plasmids

Wild type murine pikachurin plasmid was kindly provided by Dr. T. Furukawa [1]. To make a double-tagged pikachurin expression vector, a polyhistidine (6xHis) tag was first introduced to the C-terminus of pikachurin by PCR using primers FP1 (CTCGAGATGCGAACGGCTCTGCGAAAATC) and RP1 (ATCGATTTAATGATGATGATGATGATGCTTAGCCCCACAAGTGTTG). The resulting cDNA, devoid of the signal sequence (SS), was digested with XhoI/ClaI and inserted into a pCIG expression vector to generate plasmid PK-HIS. Next, a cDNA fragment encompassing pikachurin’s SS was cloned into the BamHI/ClaI sites of plasmid pCS2+MT, in which SS is immediately upstream of six copies of c-Myc. Finally, the sequence of SS and 2 copies of c-Myc were amplified by PCR and directionally cloned into the XhoI site of plasmid PK-HIS. In some experiments, a FLAG tag was substituted for c-Myc. Three N-terminal mutants (amino acids 1–354, 1–498, and 1–600) were also amplified by PCR, digested with XhoI/ClaI, and inserted into pCIG vectors. They were designated P39, P55 and P66, respectively, based on their predicted molecular weights. Dystroglycan cDNA (NM_010017 in GenBank) was amplified from mouse eye tissue and cloned into a pCIG vector. Plasmid transfection in both Y79 and QBI-HEK 293A cells was done using Lipofectamine 2000 (Invitrogen) and lasted 48–72 hrs before cells were fixed for immunocytochemistry or lysed for Western blot.

### Western Blot and Analysis

Retinal tissue and cultured cells were lysed on ice in 1xIP buffer (Sigma) supplemented with Complete Protease Inhibitor Cocktail (Roche Applied Science) and phosphatase inhibitors. Typically, 1200 µl of lysis buffer was used for one piece of human or pig retina, 150 µl for salamander retina, and 200 µl for cells cultured on a 35-mm petri dish with >85% confluence. Before lysis, cells were rinsed with PBS twice to remove possible contamination by conditioned media (CM). At the same time, CM was collected, centrifuged, and filtered through a 0.22 µm filter. To maximize the effect of protease inhibitors, tissue and cell lysates were incubated on ice with periodic vortexing for over 1 hr before proceeding to subsequent steps. Western blot was performed as previously described [7]. Briefly, protein samples were first heat-denatured in the presence of β-mercaptoethanol, separated on 3–8% Tris-acetate gel (Invitrogen) and transferred to nitrocellulose membrane. After incubation with blocking buffer (5% non-fat milk in Tris buffered saline) and primary antibodies, target proteins were detected by HRP-conjugated secondary antibodies and Luminata™ Western HRP substrate (Millipore). For quantitive analyses of pikachurin signals in the CM, Western blot results were first scanned to 16-bit Tiff files in grayscale at a resolution of 600 dpi, and the band densities were then determined using Image J software. Statistical analysis was performed with a one-way ANOVA test and a *p* value <0.05 was considered to be significant.

### Immunocytochemistry, Quantification of Fluorescence Intensity, and Live Cell Imaging

Immunocytochemistry was conducted as previously described [7]. In some experiments, cells were not permeabilized with 0.5% Triton X-100/PBS in order to detect primarily extracellular immunolabeling. After labeling, cells were mounted on slides with Fluoromount-G (SouthernBiotech) and visualized with a 63×oil objective (N.A. 1.4) on a Zeiss Axiovert 135 microscope in ApoTome optical sectioning mode. Fluorescence images, taken using AxioVision 4.5 software (Zeiss), were transferred to ImageJ v1.43 software (NIH) for fluorescence intensity measurement and color coding. The settings for imaging and processing were fixed throughout the experiments. Quantification of the fluorescence intensity was done by first subtracting the background and then measuring the average intensity from the regions of interest that were hand-traced using ImageJ. Results of measurements from multiple cell groups were then compared. A confocal microscope (LSM510; Zeiss) was used to verify key findings obtained with the ApoTome technique. For live cell imaging of pikachurin, cells were cultured on custom made coverslip-bottomed petri dishes. All antibody incubation and rinse steps were performed on ice to minimize internalization of antibodies. Imaging and analysis procedures were as above.

## Results

### Cleavage of Endogenous Pikachurin

**Figure 1:**
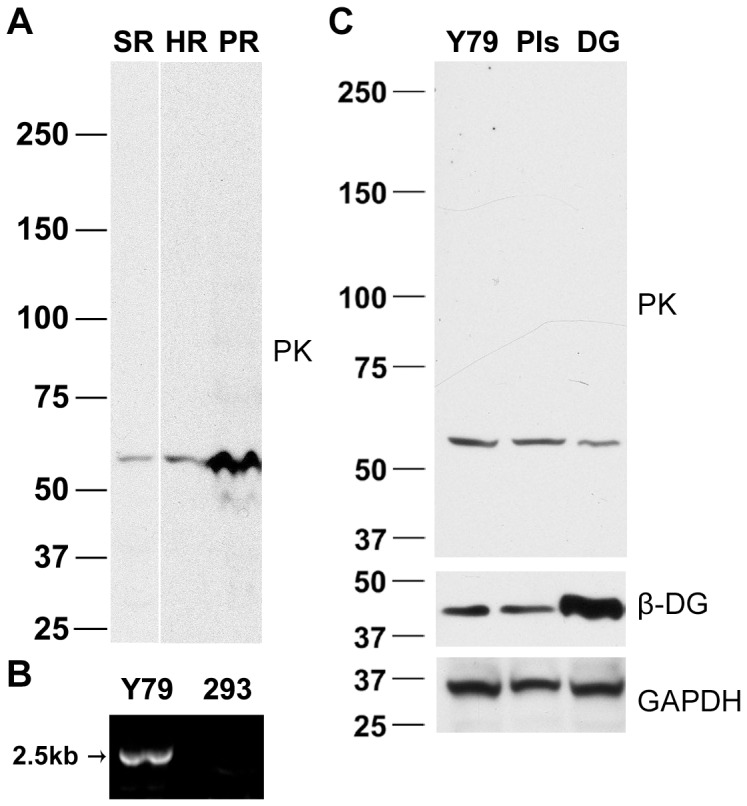
Endogenous pikachurin in retina and retina-derived cell lysates contains a ∼60-kD band, presumed to be an N-terminal fragment. (A) Western analysis of pikachurin (PK) in salamander (SR), human (HR), and pig (PR) retina. (B) Pikachurin mRNA is expressed in Y79 retinoblastoma cells. RT-PCR results showed detectable signal in Y79 cells but not QBI-HEK293 cells. (C) A similar, 60-kD, pikachurin protein fragment was detected in untreated Y79 cells, which was not affected by protease inhibition in the culture media (PIs) or overexpression of murine dystroglycan (DG).

The expression of pikachurin in murine retina has been investigated by Northern blot, *in situ* hybridization, and immunohistochemistry [1], but examination of endogenous murine pikachurin by Western blot has been, up to now, unsuccessful [8]. We began this study by examining the retinal expression of pikachurin in different species, i.e. human, pig, and tiger salamander, on Western blot. Surprisingly, a band around 60 kDa was the predominant signal for all tested adult retina tissues (Fig. 1A). The small protein is about half the size of full length pikachurin (∼110 kDa).

In order to allow for the use of molecular techniques, we also explored pikachurin in a cell line. Y79 cells were derived from human retinoblastoma and are known to express photoreceptor-specific markers [9], [10]. In untreated Y79 human retinoblastoma cells whose pikachurin mRNA expression was confirmed by RT-PCR (Fig. 1B), a 60-kD band was again found in cell lysate (Fig. 1C). Since the immunogen used to develop the pikachurin antibody was a 35-kDa N-terminal peptide (amino acids 28–354) of murine pikachurin [1], the 60-kDa band likely represents an N-terminal fragment (N-fragment) resulting from cleavage of pikachurin. These findings suggested a general processing mechanism for pikachurin in retinal tissue and retina-derived cells.

### Localization of Endogenous Pikachurin

**Figure 2:**
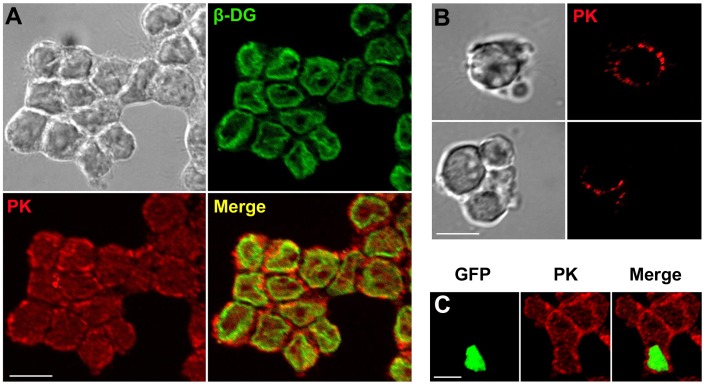
Localization of endogenous pikachurin to the cell surface in Y79 cells. (A) Immunofluorescent images of pikachurin (PK, red) and β-DG (green). Most pikachurin label is present on the extracellular side of the immunolabeling for DG, predominantly a membrane bound protein. (B) Live imaging of pikachurin in cells shows punctuate extracellular staining. (C) Overexpression of dystroglycan in transfected Y79 cells, indicated by the nuclear GFP, had no significant influence on pikachurin staining. A representative field of view is shown here, and another example can be found in Fig. S1. Bar = 10 µm.

Pikachurin is described as an extracellular protein [1], [2]. Since the Y79 cell line expressed pikachurin of a molecular weight similar to adult retina, we examined the cell culture for pikachurin localization. Biochemical techniques have indicated that pikachurin interacts with α-DG [1], [8], [11]. The possible interaction between dystroglycan and pikachurin in Y79 cells was also investigated. Y79 cells express endogenous α-DG (data not shown) and β-DG (Fig. 1C). In agreement with previous observations [12], β-DG was mainly localized to the plasmalemma in Y79 cells (Fig. 2A). Double immunolabeling of pikachurin and β-DG in fixed Y79 cells revealed strong punctuate immunofluorescence of pikachurin surrounding the ring-like signals of β-DG (Fig. 2A). In live cell imaging, uneven and punctuate labeling of pikachurin antibody was also found on the cell surface (Fig. 2B). These data, obtained using the Zeiss Apotome optical sectioning technique, were confirmed with conventional confocal microscopy (data not shown). Taken together, the results indicated that pikachurin, in particular the N-fragment, resides outside cells by attaching to either cell membrane or extracellular matrix.

We utilized a pCIG expression vector to overexpress murine dystroglycan in Y79 cells. Plasmid pCIG is an internal ribosome entry site (IRES) bicistronic expression vector which is capable of producing the target protein and a separate nuclear-type green fluorescent protein (GFP) simultaneously [13]. All GFP-positive Y79 cells had elevated levels of both α-DG and β-DG (Fig. S1) but their pikachurin immunofluorescence did not change significantly compared to non-transfected cells in the same cultures (Fig. 2C). Thus expression levels of dystroglycan and pikachurin were not correlated in these cells.

### C-terminal Fragment in Y79 Cells Expressing Exogenous Pikachurin

**Figure 3:**
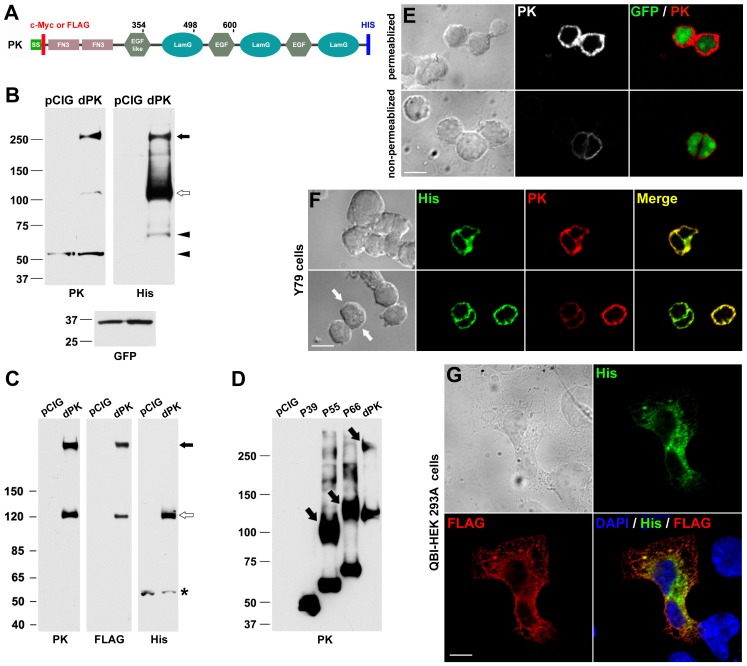
C-terminal fragment detected in Y79 cells expressing exogenous pikachurin. (A) Schematic of c-Myc/His tagged murine pikachurin (PK). SS, signal sequence; FN3, fibronectin type 3 domain; LamG, laminin G domain. The numbers indicate the end positions of three N-terminal mutants. (B) Western blots of Y79 cell lysates after transfection of double-tagged pikachurin (dPK). pCIG, pCIG-transfected controls. Note the presence of pikachurin monomer (open arrow), dimer (filled arrow), and fragments (arrowheads). PK antibody- and His antibody-labeled fragments are from the N and C-terminal respectively; PK antibody labels both endogenous and exogenous protein. (C) Western blots of QBI-HEK 293A cell lysates after transfection of dPK. There are no fragments of pikachurin but monomers (open arrow) and dimers (filled arrow) are present. A small band (*), found in both control and dPK-transfected samples, is believed to be non-specific. (D) N-terminal mutants (P39, P55 and P66) were used to transfect QBI-HEK 293A cells and their expression was confirmed on Western blot. Similar to dPK, P55 and P66 not only existed in monomers but also formed dimers (arrows). However, P39 did not dimerize indicating the loss of the site of dimerization. (E) Pikachurin (PK) immunofluorescence of dPK-transfected Y79 cells. Nuclear GFP marks transfected cells. Labeling is primarily due to overexpressed exogenous PK. Non-permeabilized immunolabeling highlights cell surface label. Bar = 10 µm. (F) Co-immunolabeling of PK and His tag in dPK-transfected Y79 cells. Immunofluorescence of PK and His tag is generally overlapping. However, some cells have relatively stronger His signals than their pikachurin counterparts (arrows). Bar = 10 µm. (G) Co-immunolabeling of FLAG and His tags in dPK-transfected QBI-HEK 293A cells. Large cell size allows the separation of signals into cytoplasmic and perinuclear regions respectively. Bar = 20 µm.

At present two pikachurin antibodies have been developed [1], [2], both against N-terminal epitopes, thus the presence of an endogenous C-terminal fragment resulting from post-translational cleavage could not be directly tested. Instead, to address this issue, we incorporated a His tag at the C-terminal of pikachurin (Fig. 3A) so that the proposed C-terminal fragment could be detected by His antibody. In addition, a c-Myc or FLAG tag was inserted immediately after the signal sequence (Fig. 3A). The expression vector containing double-tagged pikachurin was used to transfect Y79 cells. Using the pikachurin antibody, in addition to the N-fragment, a ∼120 kDa band was present on Western blots representing the full length protein of pikachurin (Fig. 3B). A band around 260 kDa, about twice the size of full length pikachurin, was also found in transfected cell lysates in significant amount (Fig. 3B). Presumably, this large band is the result of dimerization of monomers. Similar results were obtained when c-Myc instead of pikachurin antibody was used (data not shown). On Western blots using His tag antibody, the 120 kDa and 260 kDa bands were found at the same positions as on the pikachurin blot. Importantly, a small C-fragment of 65 kDa was also detected (Fig. 3B). The combined molecular weight of the two fragments, the N- and C- fragments, is very close to that of a pikachurin monomer, indicating that these two fragments are the major products of post-translational cleavage. These results support the existence of a post-translational cleavage mechanism for full length pikachurin, and suggest that an endogenous C-fragment is present.

### Pikachurin Expression in Non-retinal Cells

To test for the generality of pikachurin processing, pikachurin was expressed in QBI-HEK 293A cells, a variant of the human embryonic kidney 293 cell line. The endogenous pikachurin level in QBI-HEK 293A cells was negligible (Fig. 1B and 3C). After a FLAG/His tagged pikachurin plasmid was introduced into the cells, both monomers and oligomers were detected on Western blots (Fig. 3C). However, unlike in Y79 cells, no specific 60–65kD fragments were found in QBI-HEK 293A cells. Anti-His tag antibody picked up a ∼60-kDa band in plasmid-transfected cell samples, which is smaller than the C-fragment found in Y79 cells (Fig. 3B). This band was deemed to be non-specific since it was also present in control samples (Fig. 3C). These results not only suggest that the post-translational cleavage of pikachurin is cell type dependent, but also validate our methods of cell lysate preparation by showing that the chance of accidental pikachurin degradation during preparation was minimal. In addition, consistent with a previous report [8], our results demonstrated that pikachurin tends to form dimers when it is overexpressed.

Examination of the Western blots revealed that the monoclonal His tag antibody appeared to bind pikachurin monomers much better than dimers regardless of cell type. It is possible that the His epitope becomes less accessible when pikachurin dimerizes. We examined the site of dimerization by creating and expressing plasmids that encode N-terminal fragments of various lengths. As shown in Fig. 3D, the dimerization was independent of the C-terminal half of pikachurin but required amino acids 354–498.

### Localization of Exogenous Pikachurin

Localization of recombinant pikachurin was examined in cells showing nuclear GFP, the indicator for expression of the pikachurin plasmid (Fig. 3E). In GFP-positive Y79 cells, pikachurin signals were elevated compared to non-transfected cells and mainly cytoplasmic (Fig. 3E, upper panel). However, in a parallel experiment in which transfected cells were not permeabilized by Triton X-100, pikachurin fluorescence was also observed in GPF-positive cells albeit much weaker and was presumably on the cell surface (Fig. 3E, lower panel). Cells were imaged to detect exogenous pikachurin: the recombinant protein signal was stronger than endogenous pikachurin and thus was visualized with a much shorter exposure. Under these conditions, endogenous pikahurin, whose levels are much lower, cannot be seen. The presence of extracellular pikachurin suggests that exogenous pikachurin, similar to endogenous protein (Fig. 2), can be targeted to the cell membrane and/or extracellular matrix. Double immunolabeling in transfected Y79 cells showed broad overlap of pikachurin and His tag fluorescence, though the relative intensity (His vs. pikachurin) varied among cells or even within a single cell (Fig. 3F). It was hard to compare the subcellular localizations of the two signals, since Y79 cells are small and have limited cytoplasm. In contrast, in QBI-HEK 293A cells, which are much larger, the difference in subcellular localizations of N-terminal tag (FLAG) and C-terminal tag (His) was remarkable. His tag fluorescence was concentrated in the perinuclear area, whereas FLAG tag labeling was distributed more evenly within the cell (Fig. 3G). Given that there were no pikachurin fragments found in QBI-HEK 293A cells (Fig. 3C), the distinction in FLAG and His tag immunofluorescence may represent the difference in the subcellular localizations of pikachurin monomers and dimers, as FLAG tag antibody labeled dimers better than monomers whereas His tag antibody strongly preferred monomers (Fig. 3B and 3C).

### Pikachurin Fragments in Conditioned Media of Y79 Cells

**Figure 4:**
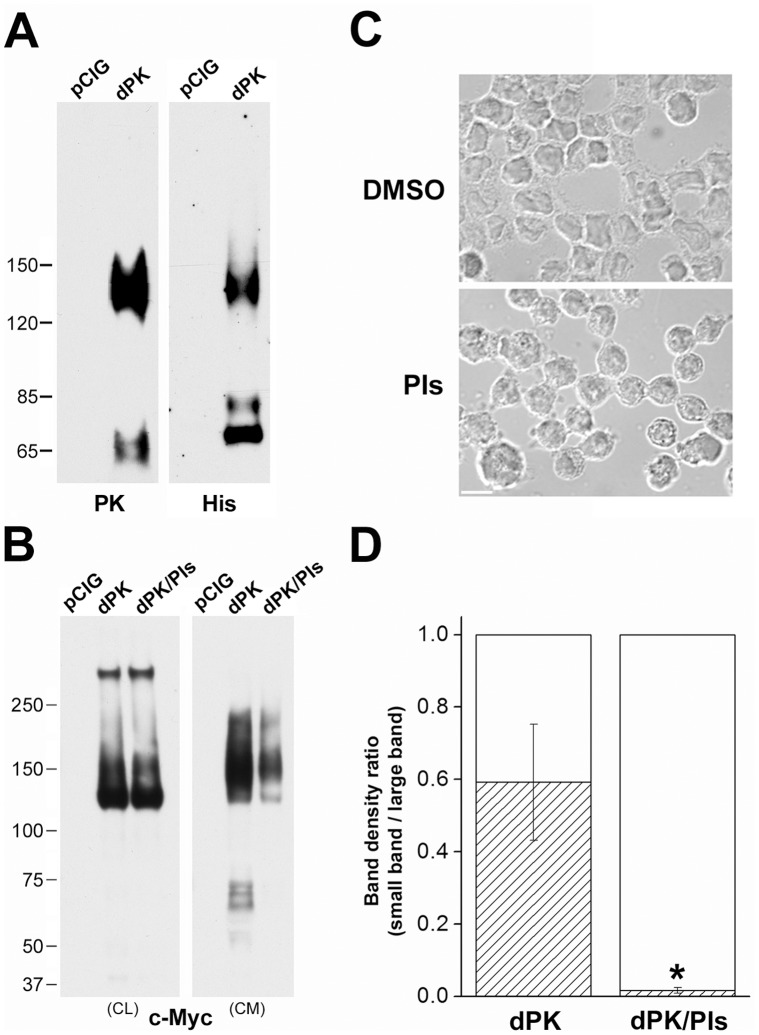
Pikachurin fragments present in the conditioned medium of Y79 cells. (A) Western blots of the conditioned medium from pCIG and dPK transfected cells. PK antibody labeled N fragments and whole protein and His antibody labeled C fragments and whole protein. (B) Inclusion of protease inhibitors in culture medium (dPK/PIs) didn’t affect the expression of dPK in the cell lysates (CL) but decreased its levels in the conditioned medium (CM). Note: c-Myc labels only recombinant protein. Short exposure reveals the high levels of recombinant pikachurin monomers and oligomers on the CL blot. Small fragments on this blot were visible with prolonged exposure but on a much darker background (data not shown). (C) Morphological changes in Y79 cells induced by incubation with protease inhibitors for 48 hrs. The solvent DMSO was diluted 1∶400 in the culture medium for control cells. Bar = 10 µm. (D) Density ratios of Western blot bands (N-fragments/monomers, Fig. 4B). N = 3 experiments. * = significant difference between protease inhibition treatment group (dPK/PIs) and control group (dPK) (p<0.05, one-way ANOVA test).

Pikachurin possesses a typical signal sequence at its N-terminus and can be exocytosed [1], [2], [8], [11]. In conditioned medium (CM) from Y79 cells transfected with the control plasmid, very little whole or cleaved endogenous protein was found (Fig. 4A). On the other hand, intense smeared bands stretching from 120 to more than 150 kDa were detected in the CM of cells expressing recombinant pikachurin on Western blots with both pikachurin and His tag antibodies (Fig. 4A). These smeared bands may represent pikachurin proteins with various degrees of glycosylation. In fact, pikachurin secreted in the conditioned medium of pikachurin-transfected 293F cells has been reported to be highly glycosylated and the glycosylation was susceptible to heparinase/heparitinase and chondroitinase [2]. Interestingly, pikachurin oligomers were found in the CM but only at very low levels, suggesting that glycosylation might interfere with the interaction between monomers. However, oligomers were found in cell lysates (Fig. 4B) repeating earlier results (Fig. 3B). Taken together, our data demonstrated that excessive pikachurin forms intracellular oligomers and monomers. The latter also accumulated in the culture media, possibly due to the saturation of post-translational processing machinery as well as extracellular binding sites. It is noteworthy that N- and C-fragments were discovered in the CM as well (Fig. 4A). But these bands appeared smeared and migrated more slowly than their counterparts in the cell lysates (Fig. 3B). It is possible that they were produced by enzymatic cleavage of glycosylated monomers. To investigate this possibility, a protease inhibitor cocktail was included in the culture media as described in Materials and Methods. Because of poor cell membrane permeability, the inhibitors had little effect on the generation of endogenous N-fragments (Fig. 1C) or the expression of recombinant pikachurin in the cell lysates (Fig. 4B). In addition, protease inhibition did not affect cell viability in our experiments, although it did induce obvious morphological changes, such as reduction in cell size and loss of contact with neighboring cells (Fig. 4C). The levels of intact pikachurin and N-fragment were reduced in the CM (Fig. 4B). This may have been the consequence of decreased pikachurin secretion. However, quantitative analysis of the amount of N-fragments in relation to the monomers (expressed as the signal ratio of N-fragment band *vs.* monomer band in the CM) revealed that in the protease inhibition treatment group the ratio is significantly lower than that of control (Fig. 4D), indicating that the reduction of N-fragments in the CM reflected not only decreased monomer secretion but more profoundly, inhibition of proteolytic cleavage. In fact, the inhibition was so complete that pikachurin N-fragments were nearly absent in the CM (Fig. 4B). These results suggest that cleavage of pikachurin can occur extracellularly.

## Discussion

In this report, we show that a recently identified retinal protein, pikachurin, is post-translationally cleaved in adult retina and human retinoblastoma Y79 cells. This modification is reminiscent of that for dystroglycan. Translated initially as a single protein with signal sequence at its N terminal and a trans-membrane domain at the C terminal, dystroglycan is trafficked into cellular membrane, where its extracellular portion is proteolytically cleaved generating two subunits (α and β). The α-dystroglycan is thus an extracellular protein which interacts with extracellular matrix [12]. Mammalian pikachurin contains an N-terminal signal sequence and has been routinely found in the culture medium of cells expressing the recombinant protein [1], [2], [8], [11]. The analogy with dystroglycan is however only partial. This current study with Y79 cells suggests that endogenous pikachurin mainly underwent cleavage instead of being secreted into the medium as a whole protein. In pikachurin-transfected cells, the cleavage generated two major fragments with a combined molecular weight approximating that of the whole protein. Our immunolabeling data indicate that endogenous N-fragments immobilized extracellularly in Y79 monolayer cell culture (Fig. 2), which is in agreement with previous findings that recombinant pikachurin is able to deposit in the extracellular matrix of differentiated myoblasts [2]. Moreover, the N-fragment of pikachurin very likely contains two type III fibronectin domains (FN3, Fig. 3A). FN3 is an evolutionary conserved protein domain that is often found in multiple repeats in the extracellular regions of various cell adhesion molecules [14]. At present, the function of the endogenous N-terminal fragments of pikachurin in the photoreceptor ribbon synapse is unknown. The presence of endogenous C-terminal fragments remain to be directly determined although cleavage of the exogenous pikachurin indicates that C-terminal fragments are possible. It is noteworthy that enzymatic digestion also took place for secreted pikachurin in conditions of overexpression (Fig. 4). The mechanisms for these cleavage processes are not clear and need further investigation.

It has been proposed that pikachurin is an adaptor protein binding to α-DG on one side of the ribbon synapse and with another partner, yet to be identified, on the other side [1]. Despite biochemical evidence demonstrating that pikachurin binds to α-DG through LamG domains at its C-terminus [1], [8], [11], the interaction between these two proteins in a more physiological setting is elusive. First, although the localization of pikachurin in the outer plexiform layer (OPL) of the retina appears to be unambiguous [1], [8], [11], convincing evidence for the presence of α-DG in the OPL is lacking. Montanaro et al. (1995) did present immunofluorescence data showing faint α-DG staining in the OPL, but that labeling was later attributed by one of the authors to anti-α-DG antibody’s cross reactivity with β-DG [15]. Second, immunohistochemical studies demonstrated co-localization of pikachurin and β-DG within the ribbon synapses [1], [8], [11], however, the distribution of α-DG and β-DG is not always overlapping [16], [17]. In our study, we have not observed any correlation among the expression of pikachurin, pikachurin fragments, and dystroglycans.

Pikachurin is a potentially useful molecule in synaptogenesis, and future studies on this protein may unavoidably involve upregulation of its expression. Thus, it should be noted that overexpressed pikachurin tends to form intracellular oligomers and accumulate as monomers in the culture medium. Both of these outcomes seem distinct from the endogenous situation in retina. Further, overexpression of double-tagged pikachurin in Y79 cells produced only a moderate increase in the signals of N-fragments on Western blot (Fig. 3B) and extracellular immunofluorescence of c-Myc (N-terminal tag, Fig. 3E), suggesting that the amount of extracellular N-fragments was dependent on the availability of as yet unidentified receptors as well as the competition between endogenous and exogenous pikachurin for these receptors.

The phenomenon of pikachurin oligomerization, first reported by us at the ARVO meeting in 2009, was later confirmed by Kanagawa et al. (2010), who also showed that the first LamG domain was critical for oligomerization. In agreement with their observations, our results indicate that the amino acid sequence from 354 to 498, which includes most of the first LamG domain, is required for pikachurin oligomerization (Fig. 3D). It is likely that oligomerization induces some conformational change in pikachurin, e.g. the C-terminus becomes less exposed, as suggested by our observations with His antibody (Fig. 3B, C). It is intriguing that very little oligomerized recombinant pikachurin was found in the conditioned media of Y79 cells. Perhaps, concealment of oligomerization sites by glycosylation could be one contributing factor. Finally, oligomerization was observed in both Y79 and HEK293 cells, suggesting it is a general capability of pikachurin and that understanding of pikachurin function in retina may require use of specific cell types.

In conclusion, our work underscores the unique role of pikachurin in retina. Not only is pikachurin highly expressed in retina, but it has tissue specific post-translational processing. The fact that most endogenous pikachurin is cleaved suggests intrinsic regulation within retinal tissue and Y79 cells. The mechanisms underlying the post-translational processing of pikachurin have yet to be determined.

## Supporting Information

Figure S1 **Overexpression of murine dystroglycan in Y79 cells.** (A, B) Simultaneous increase in the levels of α-DG (red) and β-DG (green) levels after overexpression of murine dystroglycan in Y79 cells. Two sets of representative immunofluorescent images are shown. (C) Overexpression of dystroglycan in Y79 cells, indicated by the nuclear GFP, had no significant influence on pikachurin staining. Bar = 10 µm.
(TIF)
