# Supplementary figures and images for: Cell Specific Post-Translational Processing of Pikachurin, A Protein Involved in Retinal Synaptogenesis
Source: PLoS One. 2012 Dec 4;7(12):e50552. doi: 10.1371/journal.pone.0050552 (PMC3514312; doi:10.1371/journal.pone.0050552)

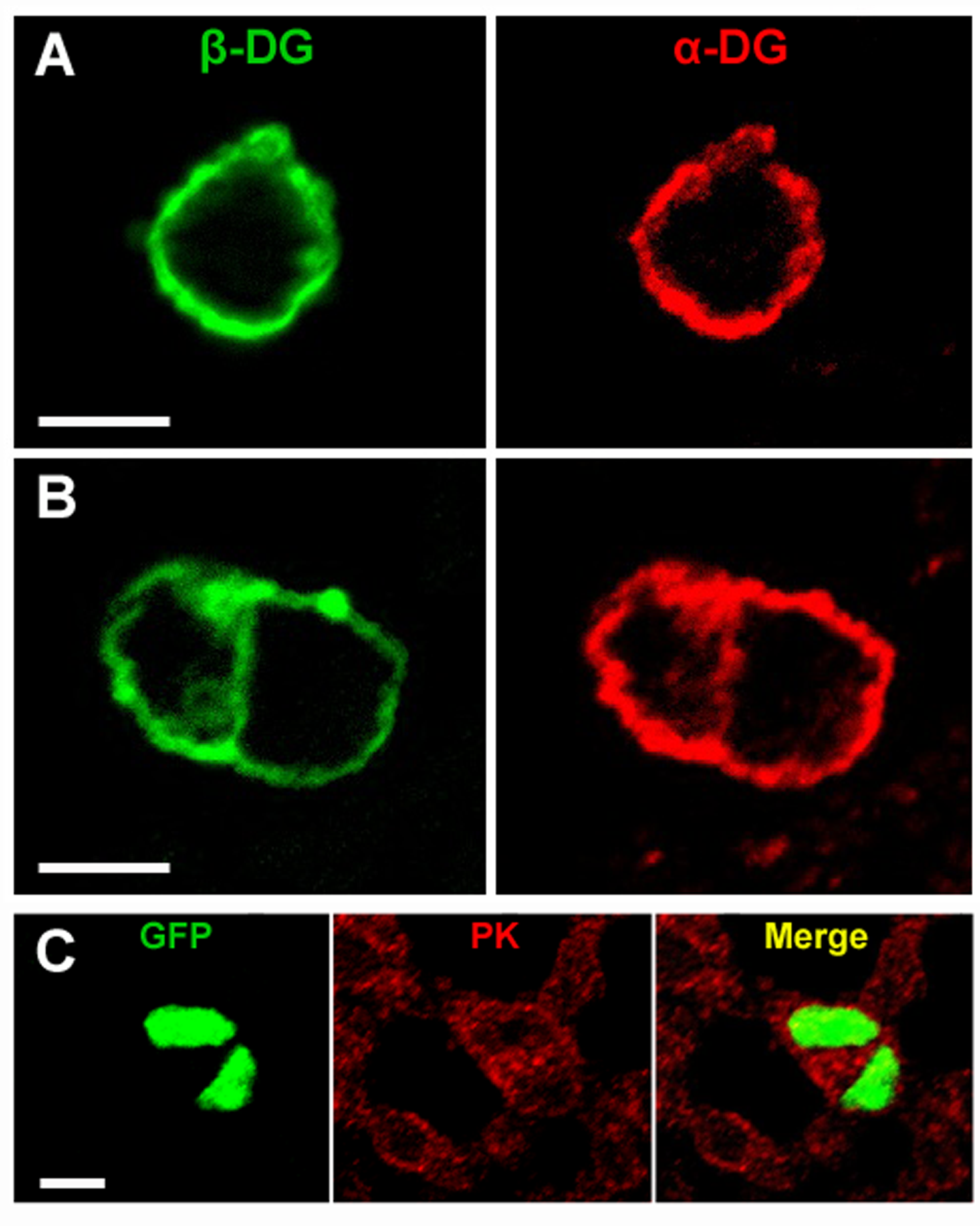

Supplement: Figure S1 — Overexpression of murine dystroglycan in Y79 cells. (A, B) Simultaneous increase in the levels of α-DG (red) and β-DG (green) levels after overexpression of murine dystroglycan in Y79 cells. Two sets of representative immunofluorescent images are shown. (C) Overexpression of dystroglycan in Y79 cells, indicated by the nuclear GFP, had no significant influence on pikachurin staining. Bar = 10 µm. (TIF) [file pone.0050552.s001.tif]
